# Supplementary material for: Chemical and structural investigation of the paroxetine-human serotonin transporter complex
Source: eLife. 2020 Jul 3;9:e56427. doi: 10.7554/eLife.56427 (PMC7470834; doi:10.7554/eLife.56427)
Supplement: Supplementary file 1. [file elife-56427-supp1.docx]

**HPLC Traces for Racemic, Scalemic and Enantioenriched Br-Piperidine Derivatives (±)-S2a, (±)-S3a, (+)-6a, (+)-7a**, **(–)-S3a, (–)-8a and (+)-S4a**

***tert-*Butyl *cis-*(±)*-*4-(4-bromophenyl)-3-(quinolin-8-ylcarbamoyl)piperidine-1-carboxylate ((±)-S2a)**

**Conditions**: Chiralpak IA 3-column, 85:15 *n*-hexane:*i*-PrOH, flow rate: 1 mL·min^–1^, 35 ºC, UV detection wavelength: 210.4 nm. Retention times: 11.9 min (3*S,*4*S* enantiomer), 17.3 min (3*R,*4*R* enantiomer).

**
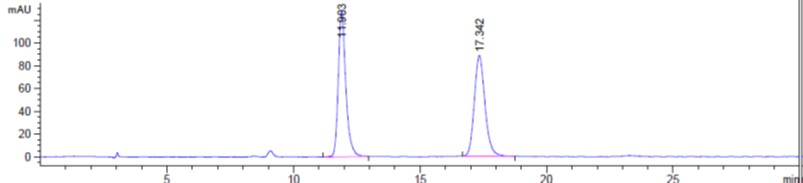
**

***tert-*Butyl (+)-(*3R,4R*)-4*-*(4*-*bromophenyl)*-*3*-*(quinolin*-*8*-*ylcarbamoyl)piperidine*-*1*-*carboxylate ((+)*-*6a)**

**
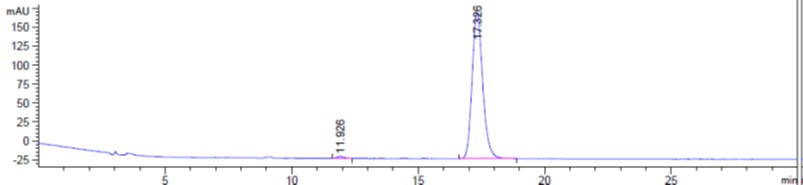
**

** *ee* = 98.2%**

***tert-*Butyl *trans-*(±)*-*4*-*(4*-*bromophenyl)*-*3*-*(quinolin*-*8*-*ylcarbamoyl)piperidine*-*1*-*carboxylate ((±)-S3a)**

**Conditions**: Chiralpak IA 3-column, 85:15 *n*-hexane:*i*-PrOH, flow rate: 1 mL·min^–1^, 35 ºC, UV detection wavelength: 254.1 nm. Retention times: 9.1 min (3*R,*4*S* enantiomer), 12.2 min (3*S,*4*R* enantiomer).

**
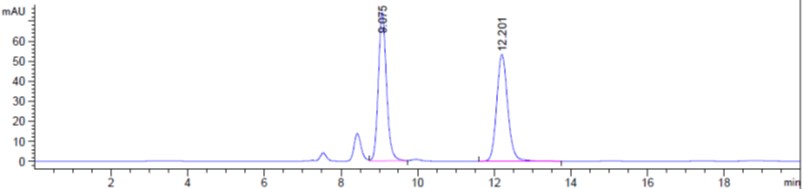
**

***tert*-Butyl (–)-(*3R,4S*)-4-(4-bromophenyl)-3-(quinolin-8-ylcarbamoyl)piperidine-1-carboxylate
((–)-S3a)**

**
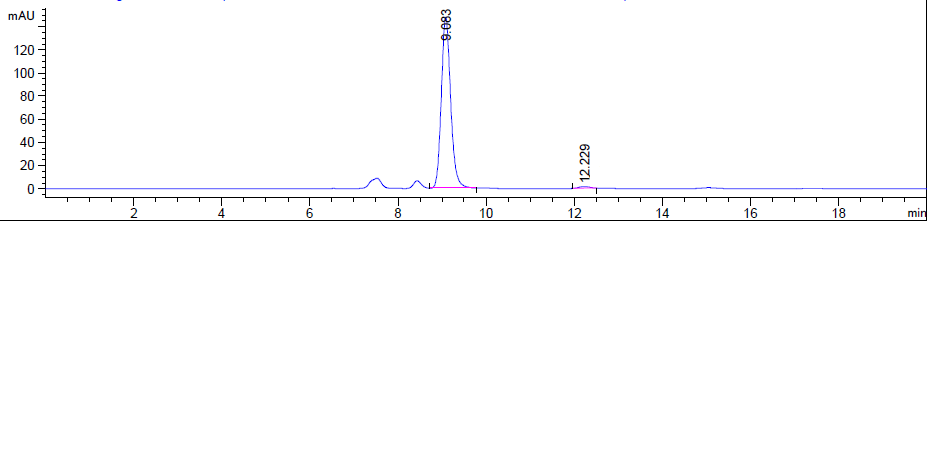
**

*** ee* 98.0%**

***tert*-Butyl (+)-(*3S,4R*)-4-(4-bromophenyl)-3-(quinolin-8-ylcarbamoyl)piperidine-1-carboxylate ((+)-7a)**

**
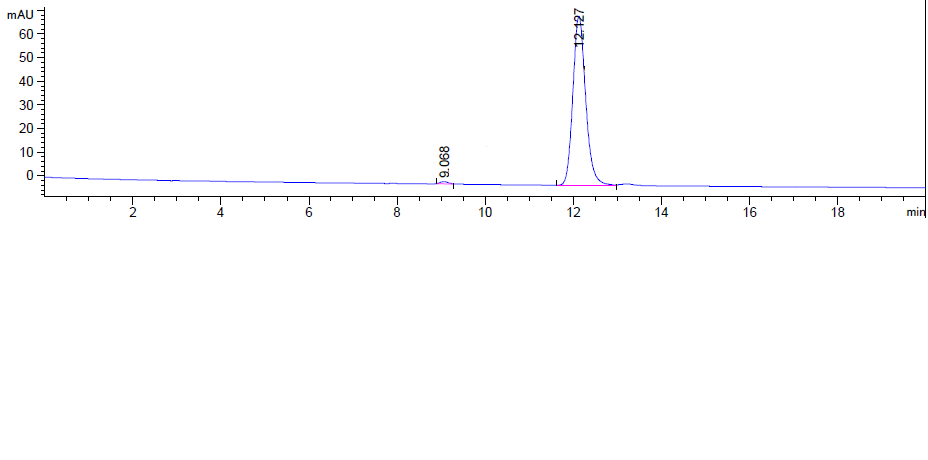
**

** *ee* = 98.4%**

**Scalemic Mixture of Enantioenriched Br-Piperidines (+)-S4a and (–)-8a**

**Conditions**: Chiralpak ID 3-column, 90:10 *n*-hexane:*i*-PrOH, flow rate: 1 mL·min^–1^, 35 ºC, UV detection wavelength: 210.4 nm. Retention times: 8.0 min (3*R,*4*S* enantiomer), 8.6 min (3*S,*4*R* enantiomer).

***tert*-Butyl (+)-(*3R,4S*)-4-(4-bromophenyl)-3-(hydroxymethyl)piperidine-1-carboxylate ((+)-S4a)**

**
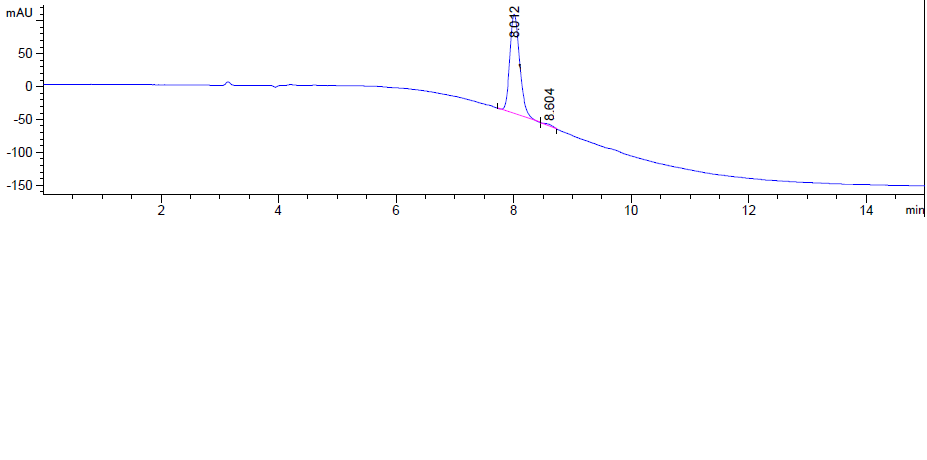
**

** *ee* = 98.1%**

***tert*-Butyl (–)-(*3S,4R*)-4-(4-bromophenyl)-3-(hydroxymethyl)piperidine-1-carboxylate ((–)-8a)**

**
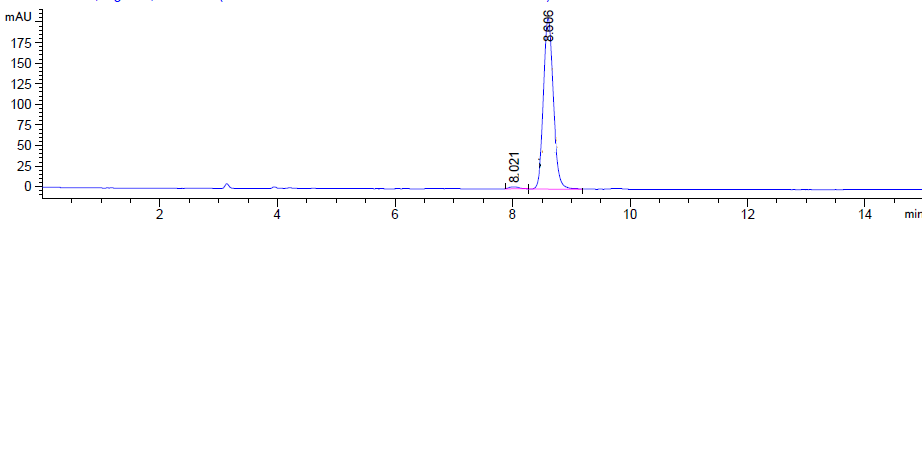
**

** *ee* = 98.1%**

**HPLC Traces for Racemic, Scalemic and Enantioenriched I-Piperidine Derivatives (±)-S2b, (±)-S3b, (+)-6b, (+)-7b**, **(–)-S3b, (–)-8b and (+)-S4b**

***tert-*Butyl *cis-*(±)*-*4-(4-iodophenyl)-3-(quinolin-8-ylcarbamoyl)piperidine-1-carboxylate ((±)-S2b):**

**Conditions**: Chiralpak IA 3-column, 85:15 *n*-hexane:*i*-PrOH, flow rate: 1 mL·min^–1^, 35 ºC, UV detection wavelength: 210.4 nm. Retention times: 12.2 min (3*S,*4*S* enantiomer), 17.7 min (3*R,*4*R* enantiomer).

**
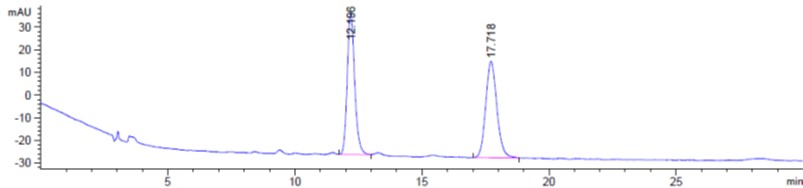
**

***tert-*Butyl (+)-(*3R,4R*)-4*-*(4*-*iodophenyl)*-*3*-*(quinolin*-*8*-*ylcarbamoyl)piperidine*-*1*-*carboxylate ((+)*-*6b**

**
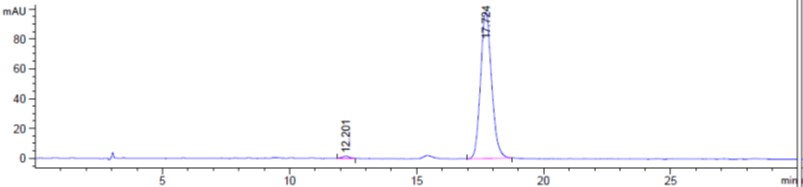
**

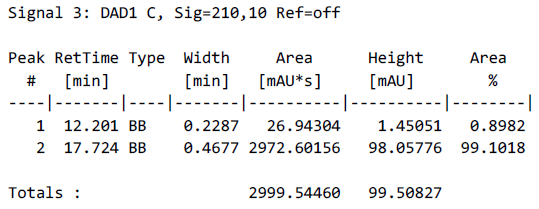
 ***ee* = 98.2%**

***tert-*Butyl *trans-*(±)*-*4*-*(4*-*iodophenyl)*-*3*-*(quinolin*-*8*-*ylcarbamoyl)piperidine*-*1*-*carboxylate ((±)-S3b)**

**Conditions**: Chiralpak IA 3-column, 85:15 *n*-hexane:*i*-PrOH, flow rate: 1 mL·min^–1^, 35 ºC, UV detection wavelength: 254.1 nm. Retention times: 9.4 min (3*R,*4*S* enantiomer), 13.3 min (3*S,*4*R* enantiomer).

**
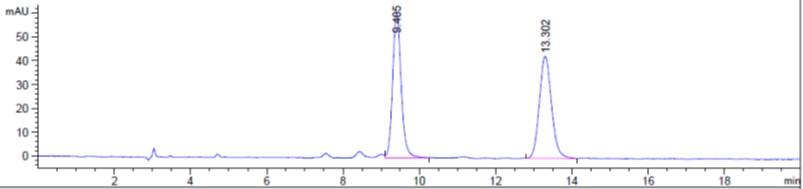
**

***tert*-butyl (–)-(*3R,4S*)-4-(4-iodophenyl)-3-(quinolin-8-ylcarbamoyl)piperidine-1-carboxylate
((–)-S3b)**


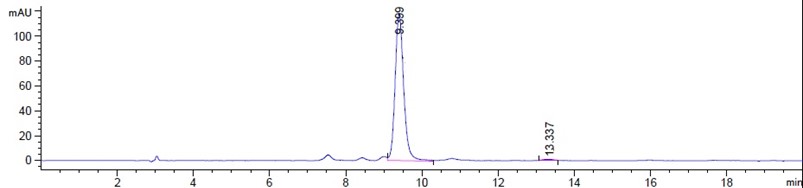

** *ee* = 98.1%**

***tert*-Butyl (+)-(*3S,4R*)-4-(4-iodophenyl)-3-(quinolin-8-ylcarbamoyl)piperidine-1-carboxylate ((+)-7b)**

**
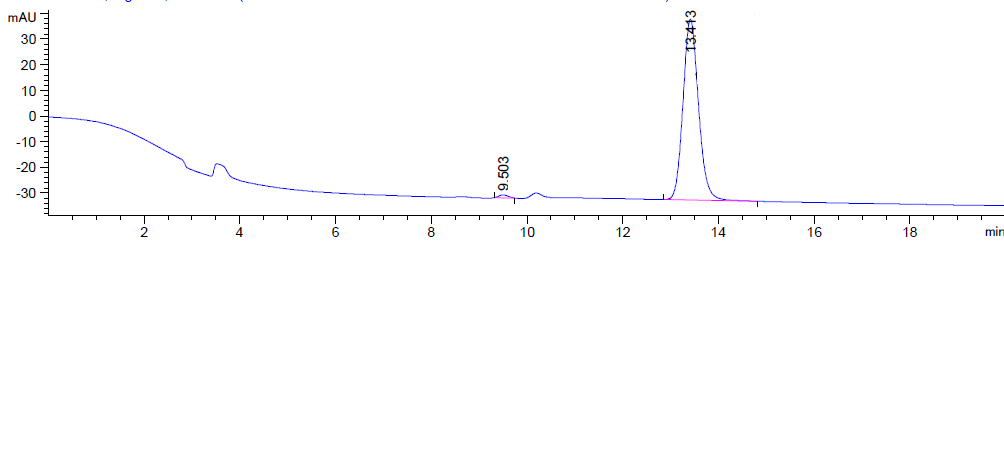
**

** *ee* = 98.0%**

**Scalemic Mixture of Enantioenriched I-Piperidines (+)-S4b and (–)-8b**

**Conditions**: Chiralpak ID 3-column, 90:10 *n*-hexane:*i*-PrOH, flow rate: 1 mL·min^–1^, 35 ºC, UV detection wavelength: 230.1 nm. Retention times: 6.7 min (3*R,*4*S* enantiomer), 7.4 min (3*S,*4*R* enantiomer).

***tert*-Butyl (+)-(*3R,4S*)-4-(4-iodophenyl)-3-(hydroxymethyl)piperidine-1-carboxylate ((+)-S4b)**

**
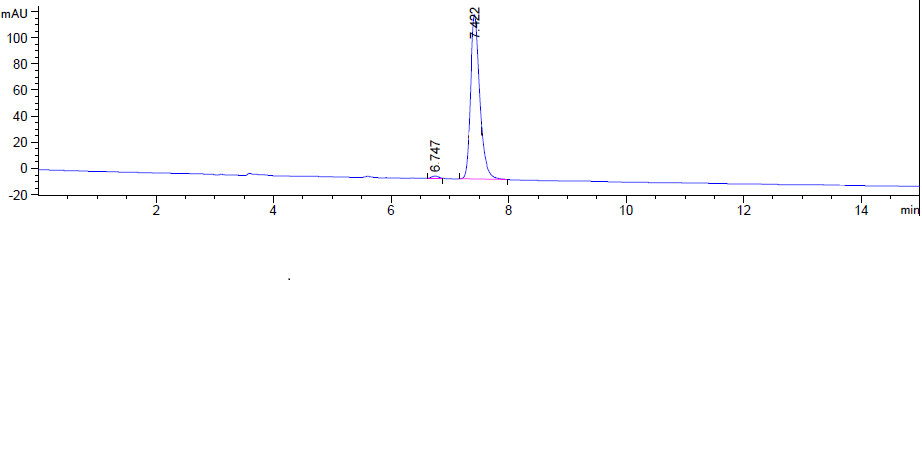
**

*** ee* = 98.1%**

***tert*-Butyl (–)-(*3S,4R*)-4-(4-iodophenyl)-3-(hydroxymethyl)piperidine-1-carboxylate ((–)-8b)**

**
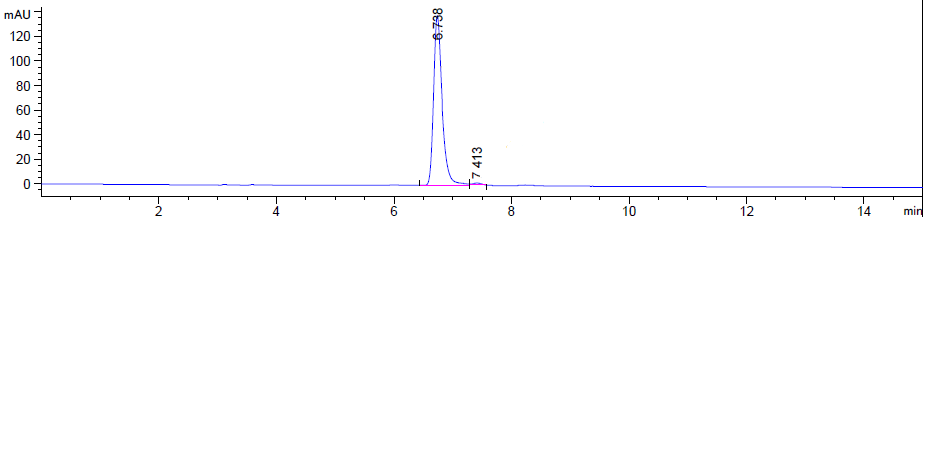
**

*** ee* = 98.0%**
