# Supplementary material for: Chemical and structural investigation of the paroxetine-human serotonin transporter complex
Source: eLife. 2020 Jul 3;9:e56427. doi: 10.7554/eLife.56427 (PMC7470834; doi:10.7554/eLife.56427)
Supplement: Supplementary file 2. [file elife-56427-supp2.docx]

**NMR Spectra for Novel Compounds**

**(±)-S2a**

^1^H NMR (500 MHz, (CD_3_)_2_SO, 373 K)

**(±)-S2a**

^13^C NMR (126 MHz, (CD_3_)_2_SO, 373 K)

**(±)-S3a**

^1^H NMR (500 MHz, (CD_3_)_2_SO, 373 K)

**(±)-S3a**

^13^C NMR (126 MHz, (CD_3_)_2_SO, 373 K)


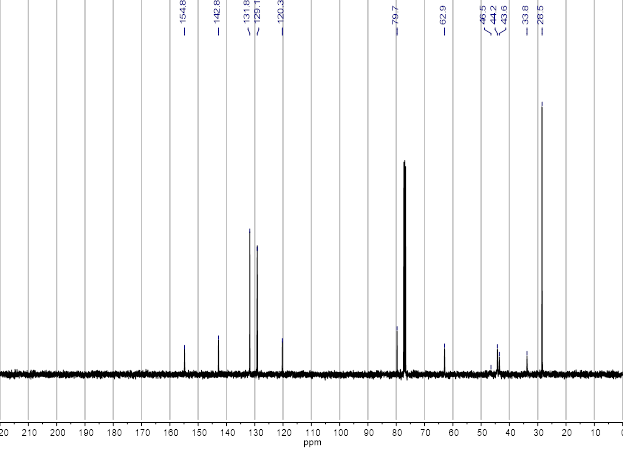


**(+)-S4a**

^13^C NMR (101 MHz, CDCl_3_)

**(+)-S4a**

^1^H NMR (400 MHz, CDCl_3_)

**(+)-S4a**

HSQC NMR (400 MHz, CDCl_3_)

**(–)-9a**

^13^C NMR (101 MHz, CDCl_3_)

**(–)-9a**

^1^H NMR (400 MHz, CDCl_3_)

**(–)-9a**

HSQC (400 MHz, CDCl_3_)

**2** ∙ HCl

^1^H NMR (400 MHz, CD_3_OD)

**2** ∙ HCl

^13^C NMR (101 MHz, CD_3_OD)

**2** ∙ HCl

COSY (400 MHz, CD_3_OD)

**2** ∙ HCl

HSQC (400 MHz, CD_3_OD)


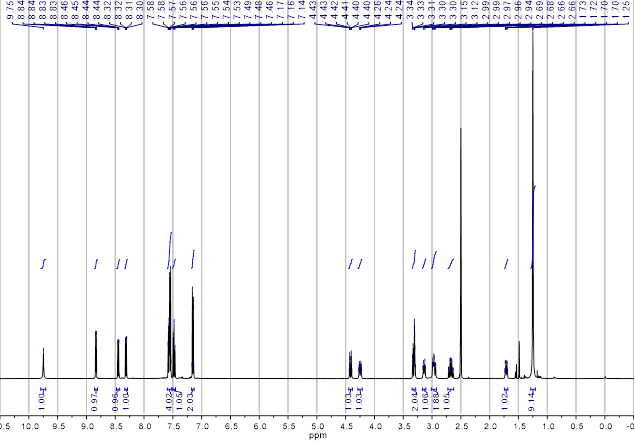

**(±)-S2b**

^1^H NMR (500 MHz, (CD_3_)_2_SO, 373 K)

**(±)-S2b**

^13^C NMR (126 MHz, (CD_3_)_2_SO, 373 K)

**(±)-S3b**

^13^C NMR (126 MHz, (CD_3_)_2_SO, 373 K)

**(±)-S3b**

^1^H NMR (500 MHz, (CD_3_)_2_SO, 373 K)

**(+)-S4b**

^13^C NMR (101 MHz, CDCl_3_)

**(+)-S4b**

^1^H NMR (400 MHz, CDCl_3_)

**(–)-9b**

^13^C NMR (101 MHz, CDCl_3_)

**(–)-9b**

^1^H NMR (400 MHz, CDCl_3_)

**3** ∙ HCl

^13^C NMR (101 MHz, CD_3_OD)

**3** ∙ HCl

^1^H NMR (400 MHz, CD_3_OD)
